# ATP Binding and Hydrolysis Properties of ABCB10 and Their Regulation by Glutathione

**DOI:** 10.1371/journal.pone.0129772

**Published:** 2015-06-08

**Authors:** Wei Qiu, Marc Liesa, Elizabeth P. Carpenter, Orian S. Shirihai

**Affiliations:** 1 Department of Medicine, Obesity and Nutrition Section, Mitochondria ARC, Evans Biomedical Research Center, Boston University School of Medicine, Boston, Massachusetts, United States of America; 2 Structural Genomics Consortium, Nuffield Department of Clinical Medicine, University of Oxford, Oxford, United Kingdom; 3 Department of Clinical Biochemistry, School of Medicine, Ben Gurion University, Beer-Sheva, Israel; University of Cambridge, UNITED KINGDOM

## Abstract

ABCB10 (ATP binding cassette sub-family B10) is a mitochondrial inner-membrane ABC transporter. ABCB10 has been shown to protect the heart from the impact of ROS during ischemia-reperfusion and to allow for proper hemoglobin synthesis during erythroid development. ABC transporters are proteins that increase ATP binding and hydrolysis activity in the presence of the transported substrate. However, molecular entities transported by ABCB10 and its regulatory mechanisms are currently unknown. Here we characterized ATP binding and hydrolysis properties of ABCB10 by using the 8-azido-ATP photolabeling technique. This technique can identify potential ABCB10 regulators, transported substrates and amino-acidic residues required for ATP binding and hydrolysis. We confirmed that Gly497 and Lys498 in the Walker A motif, Glu624 in the Walker B motif and Gly602 in the C-Loop motif of ABCB10 are required for proper ATP binding and hydrolysis activity, as their mutation changed ABCB10 8-Azido-ATP photo-labeling. In addition, we show that the potential ABCB10 transported entity and heme precursor delta-aminolevulinic acid (dALA) does not alter 8-azido-ATP photo-labeling. In contrast, oxidized glutathione (GSSG) stimulates ATP hydrolysis without affecting ATP binding, whereas reduced glutathione (GSH) inhibits ATP binding and hydrolysis. Indeed, we detectABCB10 glutathionylation in Cys547 and show that it is one of the exposed cysteine residues within ABCB10 structure. In all, we characterize essential residues for ABCB10 ATPase activity and we provide evidence that supports the exclusion of dALA as a potential substrate directly transported by ABCB10. Last, we show the first molecular mechanism by which mitochondrial oxidative status, through GSH/GSSG, can regulate ABCB10.

## Introduction

The mitochondrial inner membrane protein ABCB10 or ABCme (ATP binding cassette B10 or mitochondrial erythroid) was initially identified when studying genes up-regulated during erythroid differentiation by the transcription factor GATA-1 [1]. Consistent with this, ABCB10 was found to be essential for primitive erythropoiesis *in vivo* and for proper hemoglobin synthesis of erythroid cells [1, 2, 3]. However, ABCB10 is also expressed in non-erythroid tissues, such as the heart, which suggests a role of ABCB10 beyond hemoglobin synthesis. In this regard, inactivation of one allele of ABCB10 did not affect basal cardiac function, but markedly decreased heart recovery after acute induction of oxidative stress by ischemia-reperfusion. These cardiac defects induced by ischemia-reperfusion in ABCB10 +/- hearts were completely prevented by a 20 minutes pre-treatment with antioxidants [4]. Thus, ABCB10 plays a role protecting from increased oxidative stress in the mitochondria. Indeed, antioxidant treatments partially rescued hemoglobin synthesis in ABCB10 null erythroid cells [2]. However, the exact mechanism by which ABCB10 protects mitochondria from oxidative stress has not been identified, as the molecular entities transported by ABCB10 remain elusive.

As a member of the ABC transporter protein sub-family B, ABCB10 consists of two half transporters, each of which contains one transmembrane domain (TMD) that spans the mitochondrial inner membrane 6 times, via α helices, and one nucleotide binding domain (NBD), which binds and hydrolyzes ATP. ATP hydrolysis generates energy either for the transport of substrates or to facilitate the return of ABCB10 to the initial conformation after substrate transport [5, 6, 7]. The TMDs are highly variable in different ABC transporters and constitute the domain through which their substrates can cross the inner mitochondrial membrane. The NBDs are responsible for ATP binding and hydrolysis, and have highly conserved amino acid sequences, like the Walker A motif (GXXGXGKS/T, where X is any amino acid), the Walker B motif (hhhhD, where h is a hydrophobic amino acid) and the C-Loop motif (LSGGQ). In a typical transport process described for other ABC proteins, two ATP molecules are sandwiched between the Walker A and Walker B motifs of one monomer of ABC transporter and the C-loop motif of the other. In this way, the two NBDs form a sandwich dimer with a head to tail orientation [8] and perform the ATP hydrolysis cycle. This cycle includes 1) ATP binding, 2) ATP hydrolysis and the formation of a transition state complex in which both ADP and P_i_ remain bound, 3) release of P_i_, and 4) release of ADP [9, 10]. Studies performed in other ABC proteins have shown that the Walker A motif binds to ATP by the interaction of the oxygen atoms of the β- and γ-phosphates with the conserved amino acids lysine and glycine [11, 12]. The conserved glutamate residue immediately after the end of the Walker B motif is the catalytic base for ATP hydrolysis [13, 14]. The second glycine residue in the C-loop motif also interacts with the γ-phosphates of ATP and mutation of this residue decreases ATP hydrolysis in other ABC proteins [15, 16]. However, the importance of some of these conserved residues in ABCB10 function binding and hydrolyzing ATP has not been described before.

8-azido-ATP, an ATP analogue, has been widely used to study the ATP binding and hydrolysis in a wide variety of ABC transporters such as MDR1, CFTR, TAP or SUR1 [17, 18, 19, 20]. Upon exposure to UV light, the azido group on the 8-position of the adenine ring turns into the reactive nitrene group, which allows 8-azido-ATP to covalently and permanently tag the protein that it is bound to [21]. The labeling of the 8-Azido-ATP with biotin in the gamma phosphate allows determining the net changes in two related processes, ATP binding and hydrolysis, by quantifying the amount of biotin within the permanently tagged protein. To discern whether changes in biotin signal within the protein are caused by either a change in nucleotide binding or in ATP hydrolysis, 8-Azido-ATP labeled in the alpha phosphate with radioactive ^32^P is used. As the label is in the alpha phosphate, the radioactive signal within the permanently tagged protein will be exclusively a function of nucleotide binding. Therefore, the use of these 2 versions of 8-Azido-ATP can be used to determine changes in either nucleotide binding and/or hydrolysis.

In the present study, we used these differently labelled 8-Azido-ATP molecules to study the ATP binding and hydrolysis properties of ABCB10 within sub-mitochondrial particles, a more physiologically relevant context than reconstituted proteoliposomes [22, 6]. By using this approach, we determined and confirmed the relevance of the highly conserved Walker A lysine and glycine residues, the glutamate after Walker B and the second glycine in the C-loop in ABCB10 ATP binding and hydrolysis activity. And, more importantly, this approach revealed that oxidized and reduced glutathione are novel regulators of ABCB10 ATPase activity. Further, the successful development of this approach represents the establishment of a methodology to support and/or reject certain molecular entities as hypothetical substrates transported by ABCB10 in a more physiological context, as all the characterized substrates for ABC proteins stimulate ATP binding and/or hydrolysis. In this regard, we show that this approach provides evidence to exclude amino-levulinic acid (ALA), as a potential substrate directly transported by ABCB10.

## Materials and Methods

### Reagents

8-azido-ATP [γ] biotin and [α-^32^P] 8-azido-ATP were synthetized by and purchased from Affinity Photoprobes (Lexington, KY); delta-aminolevulinic acid (dALA), reduced glutathione (GSH), oxidized glutathione (GSSG), ATP, ADP, AMP, CTP, GTP, TTP, anti-V5 agarose affinity gel beads and ExtrAvidin-peroxidase were from Sigma (Saint Louis, MO); Rabbit anti-ABCB10 mouse antibody (epitope FFDKTRTGELINRL) was made by Research Genetics, Inc. (Huntsville, AL); Mouse anti-V5 antibody was from Invitrogen (Carlsbad, CA); Rabbit anti-porin/VDAC mouse antibody was from Abcam (Cambridge, MA).

### Plasmids

The cDNA encoding mouse ABCB10 (NCBI CCDS22766.1) was amplified by PCR and cloned into the plasmid pEF1α-V5-His (Invitrogen, Carlsbad, CA) at the restriction sites EcoRI and NotI. Five different ABCB10 mutants (Walker A: G497A, K498R; C-loop: G602V, G602D, and glutamate after Walker B: E624Q) were created based on the plasmid pEF1α-ABCB10-V5-His using the QuikChange XL mutagenesis kit (Agilent Technologies, Lexington, MA).

### Cell culture and transfection

HEK293 cells were cultured in 150 mm dishes in DMEM with 10% FBS and supplemented penicillin/streptomycin at 37°C in 5% CO_2_. HEK293 cells were co-transfected with pEF1α-ABCB10 (wild type or mutants)-V5-His and pEGFP-N1 (BD Biosciences, Franklin Lakes, NJ) (a total of 40 μg DNA in each dish, the ratio of ABCB10:EGFP 5:1) using the calcium phosphate transfection method when the cells were 70% confluent. 100% transfection efficiency was confirmed at 48 h after transfection by quantifying the percentage of GFP positive cells under the fluorescent microscope.

### Isolation of mitochondria and sub-mitochondrial particles (SMPs)

HEK293 cells were washed with PBS, scraped and re-suspended in ice-cold IBc buffer (10 mM Tris-MOPS, 1 mM EGTA/Tris, 200 mM sucrose, pH 7.4). The cell suspension was stroked with a Teflon pestle in a glass potter 35 times. The homogenate was centrifuged at 600 g for 10 min at 4°C. The supernatant was collected and re-centrifuged (2–3 times) until there was no pellet. The supernatant was then divided, put into 1.5 ml Eppendorf tubes and centrifuged at 7,000 g for 10 min at 4°C. The supernatant was discarded and the pellets (mitochondrial fraction) were re-suspended in 50 μl ice-cold IBc buffer. A cocktail of protease inhibitors (Roche, Indianapolis, IN) were added to the mitochondria and stored at -80°C [23]. For production of SMPs, mitochondria were mixed with 1 ml TS buffer (10 mM Tris HCl and 250 mM sucrose, pH 7.4) and sonicated on ice (5% output, 10 sec for 3 times, 20 sec intervals, 550 Sonic Dismembrator, Fisher Scientific, Hampton, NH). The suspension was ultra-centrifuged at 100,000 g, for 40 min, at 4°C. The supernatant was discarded and the pellets (SMPs) were resuspended with photolabelling buffer (2 mM ouabain, 0.1 mM EGTA, 3 mM MgCl_2_, and 40 mM Tris-HCl (pH 7.5)) [24, 25]. SMPs were stored at -80°C.

### 8-azido-ATP photolabeling and immunoprecipitation

The two different versions of 8-Azido-ATP (labeled in [γ]-biotin or [α-^32^P]) were purchased from Affinity Photoprobes (Lexington, KY). Eighty μg of SMPs were incubated on ice for 5 min with 50 μM 8-azido-ATP [γ] biotin or [α-^32^P] 8-azido-ATP in the presence or absence of various concentrations of dALA, GSH, GSSG, or different nucleotides. After that, the ice-cold reactions were irradiated with UV light (254 nm) for another 5 min. The reaction volume was 25 μl. After UV irradiation, each sample was mixed with 200 μl RIPA buffer with protease inhibitors (50 mM Tris-HCl (pH 7.5), 150 mM NaCl, 1 mM EDTA, 1% NP-40, 0.1% SDS, 0.5% sodium deoxycholate) and put on ice for 30 min to solubilize the mitochondrial membranes. Then the samples were centrifuged at 16,000 g for 30 min at 4°C. The supernatant was collected and mixed with 30 μl PBS-washed anti-V5 agarose affinity gel beads. The mixture was rotated at 4°C overnight. Then the samples were spun down at 5,000 rpm for 5 min at 4°C. The supernatant was discarded and the ABCB10-bound anti-V5 agarose affinity gel beads were washed 4 times with 500 μl RIPA buffer. After the final wash, 10 μl buffer above the beads was left. The beads were mixed with SDS-PAGE 4x sample buffer containing 8% SDS and 8% β-mercaptoethanol to make a final volume of 50 μl and heated at 100°C for 10 min. The biotin label is resistant to β-mercaptoethanol and thus to thiolysis. The beads were spun down and the supernatant was collected for Western blotting or autoradiography. The crosslinking procedure exclusively traps molecules of 8-Azido-ATP and 8-Azido-ADP that are bound to the protein nucleotide binding domains. The decrease in biotin signal, as a result of higher ATP hydrolysis activity, is a consequence of increased ADP molarity and thus more ABCB10 molecules bound to ADP (without biotin) at the time of crosslinking. At the same time, a decrease in biotin signal can be a result of decreased nucleotide binding. To discern between these two possibilities, nucleotide binding capacity is determined by the amount of radioactive signal within the protein coming from 8-Azido-ATP [α-^32^P]. As the label is in the alpha phosphate, hydrolysis will not result in the removal of the label and thus radioactive signal after crosslinking will only be dependent on nucleotide binding.

### Western blotting and autoradiography

Twenty microliters samples were loaded for SDS-PAGE analysis using 10% Bis-Tris gel (Invitrogen, Carlsbad, CA). Proteins were transferred onto PVDF membranes (Invitrogen, Carlsbad, CA). Protein expression was detected using corresponding antibodies and the biotin-labeled γ-phosphate was detected using ExtrAvidin-peroxidase. Images were taken using the Fuji LAS-4000 biomolecular imager (GE Healthcare, Pittsburgh, PA). For detection of α-^32^P, the PAGE gel was dried up using the slab dryer (Bio-Rad Model 443, Hercules, CA), autoradiography was performed using the Kodak x-ray films. Images were developed 6 days later.

## Results

### Specific dose dependent photolabeling of ABCB10 NBD with 8-azido-ATP [γ] biotin

**Fig 1:**
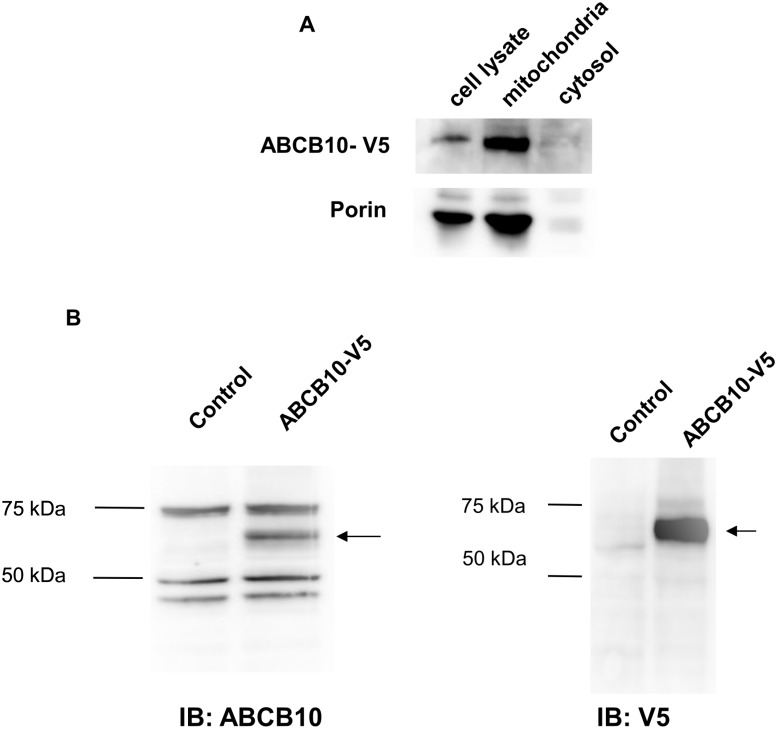
ABCB10-V5 expression and detection in intact mitochondria and SMPs. **A)** Representative Western blot analysis of over-expressed murine ABCB10-V5 in HEK293 cells after loading 20 ug of protein from total cell lysates, isolated mitochondria or cytosol in a reducing SDS-PAGE gel. ABCB10 (aprox. 65kD) was detected using anti-V5 antibody (1:5000). Detection of mitochondrial outer-membrane protein porin was used as a control for mitochondrial protein content. **B)** ABCB10-V5 expression in sub-mitochondrial particles (SMPs) obtained from isolated mitochondria from HEK293 cells transfected with empty vector (Control) or with ABCB10-V5 encoding construct. ABCB10 was detected by Western blot using anti-ABCB10 polyclonal antibody (1:2000, showing 3 unspecific bands) (left) and anti-V5 (1:5000) (right) antibody (shown by arrows).

To generate an experimental model in which ABCB10 ATP binding and hydrolysis can be tested in a more physiological context than purified ABCB10 protein within proteoliposomes, sub-mitochondrial particles (SMPs) harboring ABCB10 were prepared using mitochondria isolated from HEK293 cells over-expressing ABCB10 (Fig 1A). SMPs are mostly composed of re-sealed mitochondrial inner membranes, a portion of which are sealed inside out [26]. Since ABCB10 has been shown to have its NBD in the matrix, we expect that in inside-out vesicles the NBDs of ABCB10 are exposed to the incubation media. ABCB10 was successfully detected in SMPs by Western blotting using the antibody against either ABCB10 or V5 tag (Fig 1B). This result suggests that SMPs preparation did not lead to ABCB10 solubilization, degradation or significant C-terminal cleavage. SMPs preparations can be used to determine basal ATP binding and hydrolysis activity, which is defined as the activity of ABC proteins in the absence of their soluble substrates.

**Fig 2:**
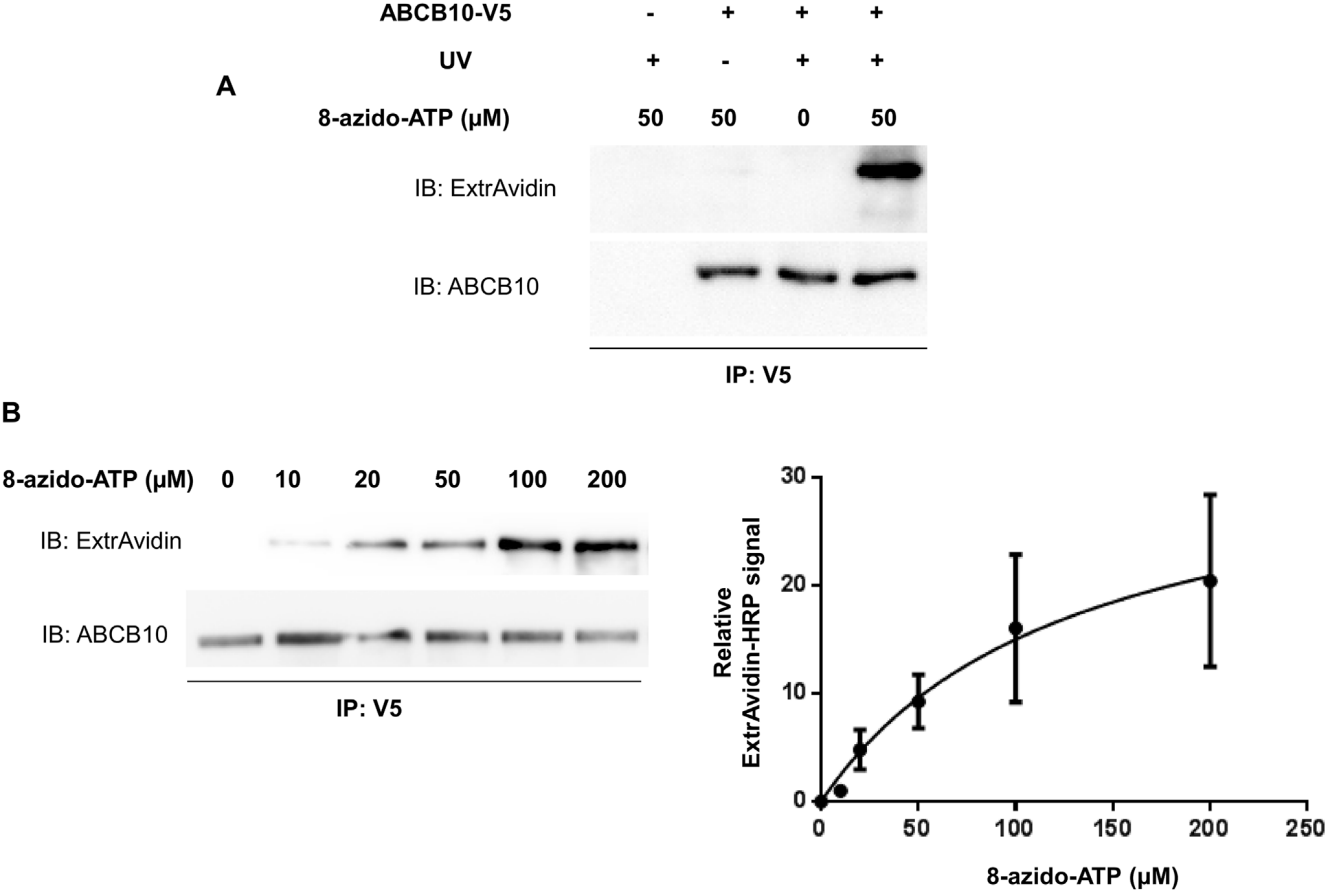
Photolabeling and ATP binding characteristics of ABCB10 using 8-azido-ATP [γ] biotin. A) SMPs (80 μg protein) from Control (-) or ABCB10-V5 (+) transfected cells were incubated with 0 or 50 μM of 8-azido-ATP [γ] biotin on ice for 5 min, treated with (+) or without (-) UV light for another 5 min and followed by IP with anti-V5 agarose affinity gel beads. After SDS-PAGE of the IP, membranes were blotted with ExtrAvidin-HRP (1:1000) or anti-ABCB10 antibody (1:2000). **B)** Representative Western blot shows that 8-azido-ATP [γ] biotin binding to ABCB10 was dose-dependent. Michaelis-Menten curve obtained from Western blot analyses is shown, with a Km value of 128.3 μM. Three independent experiments were conducted and data are shown as mean ± SEM.

The basal ATP binding and hydrolysis capacity of ABCB10 were determined by incubating SMPs with an ATP analog, 8-azido-ATP [γ] biotin, followed by UV light irradiation. UV irradiation promotes the generation of a covalent bond between the 8-azido-ATP [γ] biotin and the NBD of ABCB10. As the biotin is located in the γ-phosphate position, ATP hydrolysis activity of ABCB10 is able to remove the biotin label. Therefore, the amount of biotin in ABCB10 immuno-purified from SMPs is dependent on the net of two opposing processes at the ATP binding cassette domain, ATP binding and ATP hydrolysis by ABCB10. Successful biotin labeling of ABCB10 was detected in immuno-precipitated V5–tagged ABCB10 by Western blotting using ExtrAvidin-HRP (Fig 2A). ABCB10 pull down by immuno-capture from the SMPs is essential to specifically detect biotin labelling exclusively in ABCB10, as SMPs contains multiple mitochondrial proteins with functional NBD domains, which can also be specifically labeled. ABCB10 pull down from SMPs was quantified by Western blot detection of ABCB10, using an anti-ABCB10 antibody blot in the same samples labeled with 8-azido-ATP [γ] biotin. Biotin signal in ABCB10 increased in a dose-dependent manner and it saturated at higher 8-azido-ATP [γ] concentrations (Fig 2B). This dose-dependent increase revealed a Michaelis-Menten curve with a Km value of 128 μM 8-Azido-ATP. This affinity is within the expected range for ABC proteins and within the same range of purified ABCB10 into proteo-liposomes (Km 180–300 μM, depending on the detergent used) [6], suggesting that the biotin and the azide group does not negatively alter the ability of the modified nucleotide to bind ABCB10.

### Competition of 8-azido-ATP [γ] biotin binding with different nucleotides

**Fig 3:**
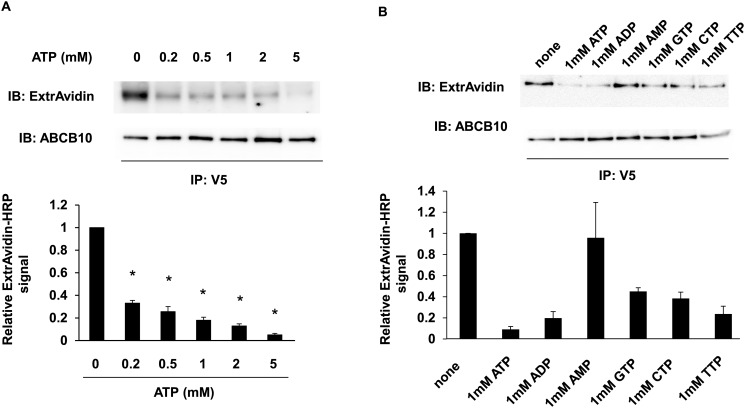
Unlabeled ATP, ADP, GTP, CTP and TTP compete with 8-azido-ATP for ABCB10 binding. A) Increasing concentrations of unlabeled ATP decreased 8-azido-ATP [γ] biotin photolabeling detected with Extravidin-HRP, indicating that the 8-Azido-ATP binding to ABCB10 is specific. Bar graphs show the relative ExtrAvidin-HRP signal per 8-azido-ATP concentration (μM). Three independent experiments were conducted and data are shown as mean ± SEM. *p<0.05 vs. signal in 0 mM ATP treatment. B) ATP and ADP had the strongest competition with 8-azido-ATP [γ] biotin to bind to ABCB10. GTP, CTP and TTP could also compete with 8-azido-ATP [γ]biotin for ABCB10 binding but to a lower extent. AMP did not compete for binding, as expected for an ABC protein. A representative Western blot analysis is shown and bar graphs represent mean ± SD from independent competition experiments.

In ABC transporters, ATP binding and hydrolysis are coupled to substrate transport. In a typical ATP hydrolysis cycle, the bound ATP is hydrolyzed and γ-phosphate is released. At that stage, ADP occupies the ATP binding site and later it is replaced by another ATP molecule allowing the next hydrolysis cycle to take place [27, 28, 29]. As a result, molecules that can compete for the ATP binding site are expected to reduce 8-Azido binding. Thus, to test for the specificity of 8-Azido-ATP ABCB10 [γ] biotin labeling, we performed competition experiments in the presence of increasing concentrations of non-labeled ATP and found that ABCB10 8-Azido-ATP ABCB10 [γ] biotin photolabeling was decreased (Fig 3A). We confirmed that ADP competes with 8 azido-ATP [γ] biotin for binding to a similar extent as ATP (Fig 3B). These results confirmed that 8-azido-ATP [γ] biotin binding by ABCB10 NBDs was specific and not a consequence of an unspecific interaction of the UV-activated nitrene group with other residues of ABCB10. We used this rationale to test for other nucleotide binding candidates. AMP, unlike ATP and ADP, did not compete and thus did not change the biotin signal detected in immuno-precipitated ABCB10 (Fig 3B). On the other hand, GTP, CTP and TTP could compete with ATP, but their effect was milder than that of ATP and ADP (Fig 3B). This competition by GTP, CTP and TTP shows that ABCB10 NBD behaves in a similar manner than other ATP/nucleotide binding proteins, as expected [30]. Altogether, these data show that ATP is the main nucleotide bound and hydrolyzed by ABCB10 to provide the energy required for the transport process.

### The effects of mutations in the conserved structures of NBD on 8-azido-ATP [γ] biotin photolabeling

**Fig 4:**
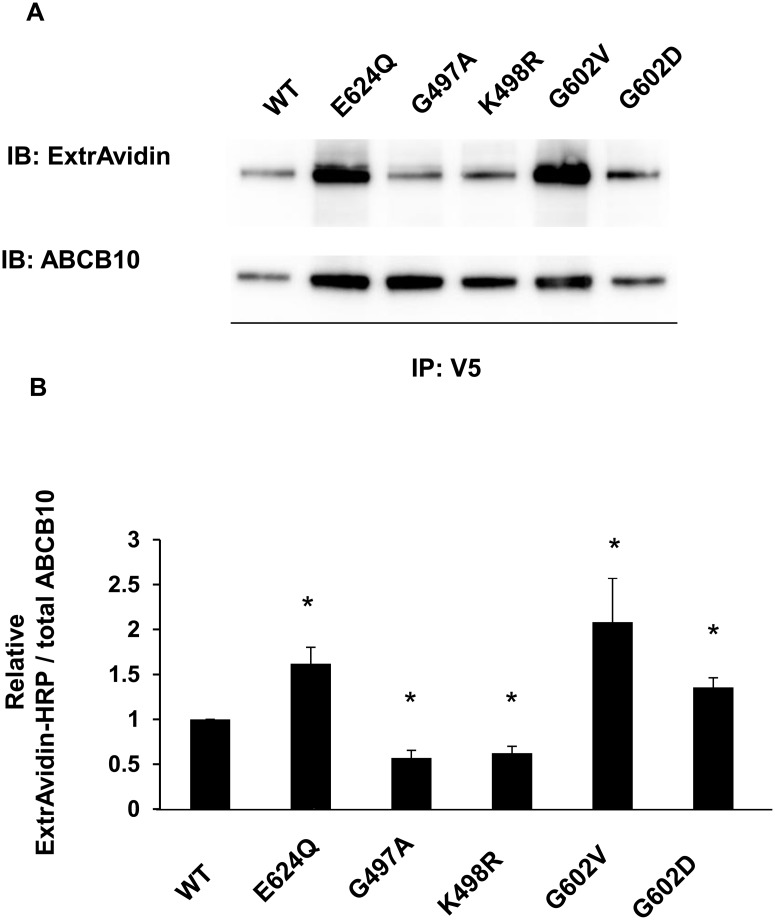
Mutations in ABCB10 NBD changed 8-azido-ATP-[γ]-biotin photolabeling according to their expected effects on ATP binding and/or hydrolysis. A) Representative Western blot analysis of SMPs from HEK293 cells transfected with wild type murine ABCB10 (WT) and different mutants (E624Q in Walker B; G497A in Walker A; K498R in Walker A; G602V in Loop C; G602D in Loop C) were photolabeled with 8-azido-ATP [γ] biotin detected with ExtrAvidin-HRP. Total ABCB10 pulled down in the V5-immunoprecipitate was detected by Western blot using a polyclonal ABCB10 antibody. B) Relative 8-azido-ATP [γ] biotin photolabeling intensity, which is consequence of both ABCB10 ATP binding and hydrolysis activity, was normalized to ABCB10 total levels, considering the different ABCB10 expression levels in different mutants. The values are shown relative to wild type ABCB10. Mutations in Walker A expected to primarily affect ATP binding decreased relative Extravidin-HRP signal by ~40%, while mutations expected to selectively affect hydrolysis but not binding (Walker B and Loop C) increased the signal. The bar graph shows the relative ExtrAvidin-HRP signal. Three independent experiments were conducted and data are shown as mean ± SEM. *p<0.05 vs. signal in wt ABCB10.

To test the functional relevance of conserved amino acid residues in the NBDs of ABCB10, we studied the effects of their mutation on 8-azido-ATP [γ] biotin photolabeling. These specific mutations have been reported to affect the ATP binding and/or hydrolysis of other ABC transporters. They include mutations in the conserved glycine and lysine residues of the Walker A motif (conversion of GPSGSGKST to GPSGSAKST, and conversion of GPSGSGKST to GPSGSGRST) [11, 12]. This set of mutations was shown to primarily affect ATP binding and concomitantly hydrolysis in numerous ABC proteins, demonstrating their conserved function among different proteins. Another set of mutations were performed in the conserved glycine of the C-loop motif (conversion of LSGGQ to LSGVQ, and conversion of LSGGQ to LSGDQ) [15, 16, 31] and in the conserved glutamate immediately after the Walker B motif (conversion of ILLLDE to ILLLDQ). Previous studies in other ABC transporters show that this set of mutations affects ATP hydrolysis activity, but not ATP binding in other ABC proteins [13, 32, 33]. Our data show that the set of mutants in the Walker A motif caused nearly 50% reduction in biotin signal, which could be likely explained by the reduced 8-azido-ATP [γ] biotin binding (Fig 4). The other 3 mutants near Walker B and in the C-loop motifs, which in other ABC transporters were shown to affect ATP hydrolysis but not binding, increased ABCB10 photo-labeling affinity by 50–100%. This increase was expected, as inhibition of 8-azido-ATP [γ] biotin hydrolysis would prevent the release of the γ-phosphate labeled with biotin (Fig 4). Thus, these changes in 8-Azido-ATP [γ] biotin labeling caused by these mutations are consistent with the expected and conserved role of these residues in ATP binding and hydrolysis within the Walker A, B and C-loop motifs. Consistent with this, a recent study confirmed these results, as the same mutation of glutamate to glutamine immediately after the Walker B motif (E624Q), in the purified and reconstituted human ABCB10 protein, completely inhibits ATP hydrolysis activity measured as phosphate release rates in this case [6].

### The 8-azido-ATP approach reveals glutathione as a regulator of ABCB10 ATP binding and hydrolysis activity

**Fig 5:**
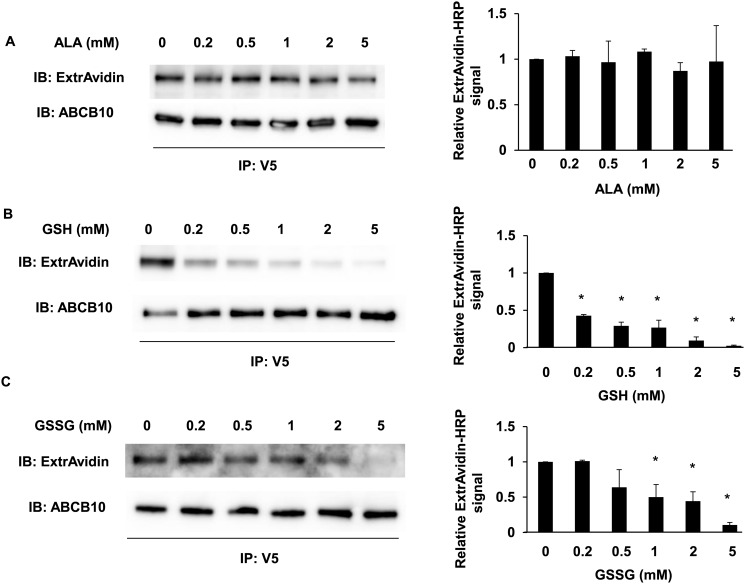
d-Amino-levulinic acid (d-ALA) does not alter 8-azido-ATP-[γ]-biotin photolabeling in ABCB10, whereas GSH and GSSG do. A) Increasing concentrations of delta-aminolevulinic acid (d-ALA) did not change the photolabeling of ABCB10 by 8-azido-ATP [γ] biotin. Representative Western blot analyses are shown. The bar graph shows the relative intensity of ExtrAvidin-HRP as mean ± SD of independent experiments. B) and C) Increasing concentrations of GSH or GSSG decreased the biotin signal during the photolabeling of ABCB10 by 8-azido-ATP [γ] biotin. The bar graph shows the relative ExtrAvidin-HRP signal. Three independent experiments were conducted and data are shown as mean ± SEM. *p<0.05 vs. signal in 0mM GSH or GSSG treatment.

The presence of substrates increases ATP binding and/or hydrolysis rates because ABC-mediated transport is activated. Since ABCB10 has been shown to be essential for hemoglobin production and erythroid differentiation [1, 2, 3], it could potentially mediate the transport of the heme biosynthesis intermediates across the mitochondrial inner membrane. SLC25A38 has been suggested to be the mitochondrial exporter of the heme precursor delta-aminolevulinic acid (dALA). Given that direct ALA transport by SLC25A38 has not been shown, it was hypothesized that ABCB10 could be directly mediating dALA export. This hypothesis was recently supported by the finding that dALA supplementation could rescue some defects caused by partial ABCB10 downregulation in cardiac cell lines [34]. Thus, we tested whether dALA could behave as an ABCB10-exported substrate, by studying whether it could activate ATP hydrolysis and thus decrease the biotin labeling in a concentration-dependent manner. Using increasing concentrations of dALA, we found that dALA did not cause any change in ABCB10 biotin levels (Fig 5A). These results suggest that dALA does not stimulate ABCB10 ATP hydrolysis activity and thus it might not be a direct substrate exported by ABCB10, at least in non-erythroid sub-mitochondrial particles. In this regard our previous studies in heart and blood suggest a role for ABCB10 in ROS handling and protection from oxidative stress instead of ALA export. Therefore, it is possible that ABCB10 activity might be regulated by the mitochondrial oxidative status/redox state. Mitochondrial GSH/GSSG levels (reduced glutathione/oxidized glutathione) and gluthathione peroxidases are essential to prevent toxicity caused by an acute increase in ROS production and oxidative stress in the mitochondrial matrix [35]. At the same time, glutathionylation of proteins can mediate some of the adaptive responses to oxidative stress and thus to changes in the mitochondrial GSSG/GSH ratio. Therefore, we hypothesized that GSSG/GSH ratio could be an acute modulator of ABCB10 activity and thus of its antioxidant function. We hypothesized that conditions of high GSSG/GSH in the matrix, associated with increased oxidative stress, would activate ABCB10 and its ATP hydrolysis activity. Thus, we examined the effect of glutathione (reduced or oxidized) on biotin labeling of ABCB10. In the presence of increasing concentrations of either GSH or GSSG, ABCB10 biotin labeling was reduced (Fig 5B and 5C).

**Fig 6:**
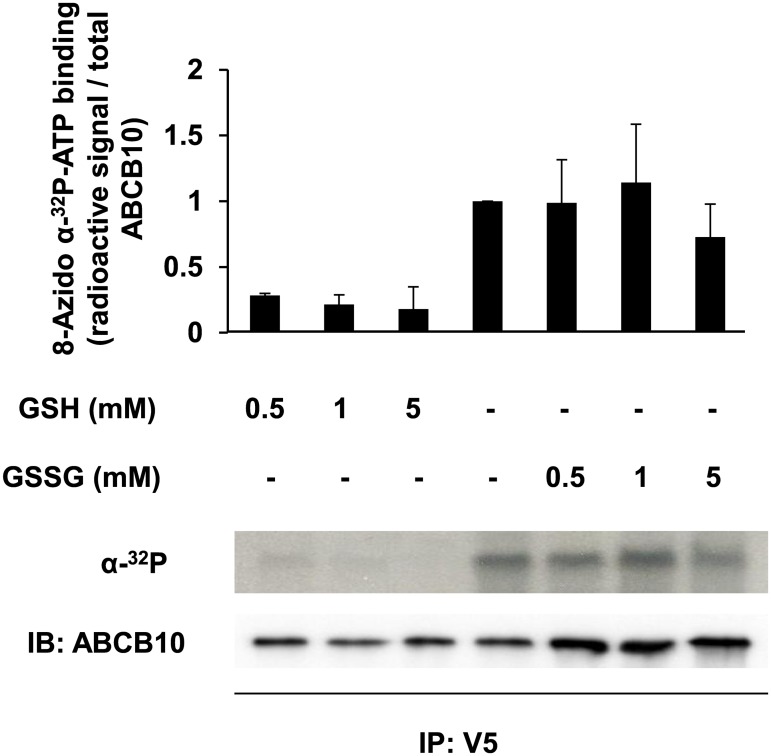
GSH, but not GSSG, specifically decreases ABCB10 ATP binding activity. A representative autoradiography image and the quantification shown in the bar graph demonstrate that GSH decreased [α-^32^P] 8-azido-ATP signal in ABCB10 from SMPs in a dose dependent manner, whereas GSSG did not. Bar graphs represent mean ± SD from independent experiments.

In marked contrast with the changes in ABCB10 biotin labeling caused by mutations in functionally conserved residues within the NBD, it is not feasible to draw any conclusion on the mechanism behind changes in ABCB10 biotin labeling caused by GSSG and GSH treatment. The main reason is that the biotin label is located in the γ-phosphate of the 8-Azido-ATP molecule, meaning that decreased ABCB10 biotin labeling could be explained by either increased ATP hydrolysis activity or decreased ATP binding. To test specific ABCB10 ATP binding activity and discern between these two events, we used [α-^32^P] 8-azido-ATP. The advantage of labeling the α-phosphate is that it is not removed by ABCB10 ATP hydrolysis activity. As a consequence, potential changes in the levels of radioactive signal in immuno-precipitated ABCB10 can only be explained by changes in ATP binding. Consistent with our hypothesis, the increasing concentrations of oxidized glutathione (GSSG) did not change the radioactive label in ABCB10. On the other hand, reduced glutathione (GSH) decreased ABCB10 radioactivity (Fig 6). These data suggest that GSSG triggered 8-azido-ATP hydrolysis by ABCB10, while GSH reduced 8-azido-ATP binding to ABCB10.

**Fig 7:**
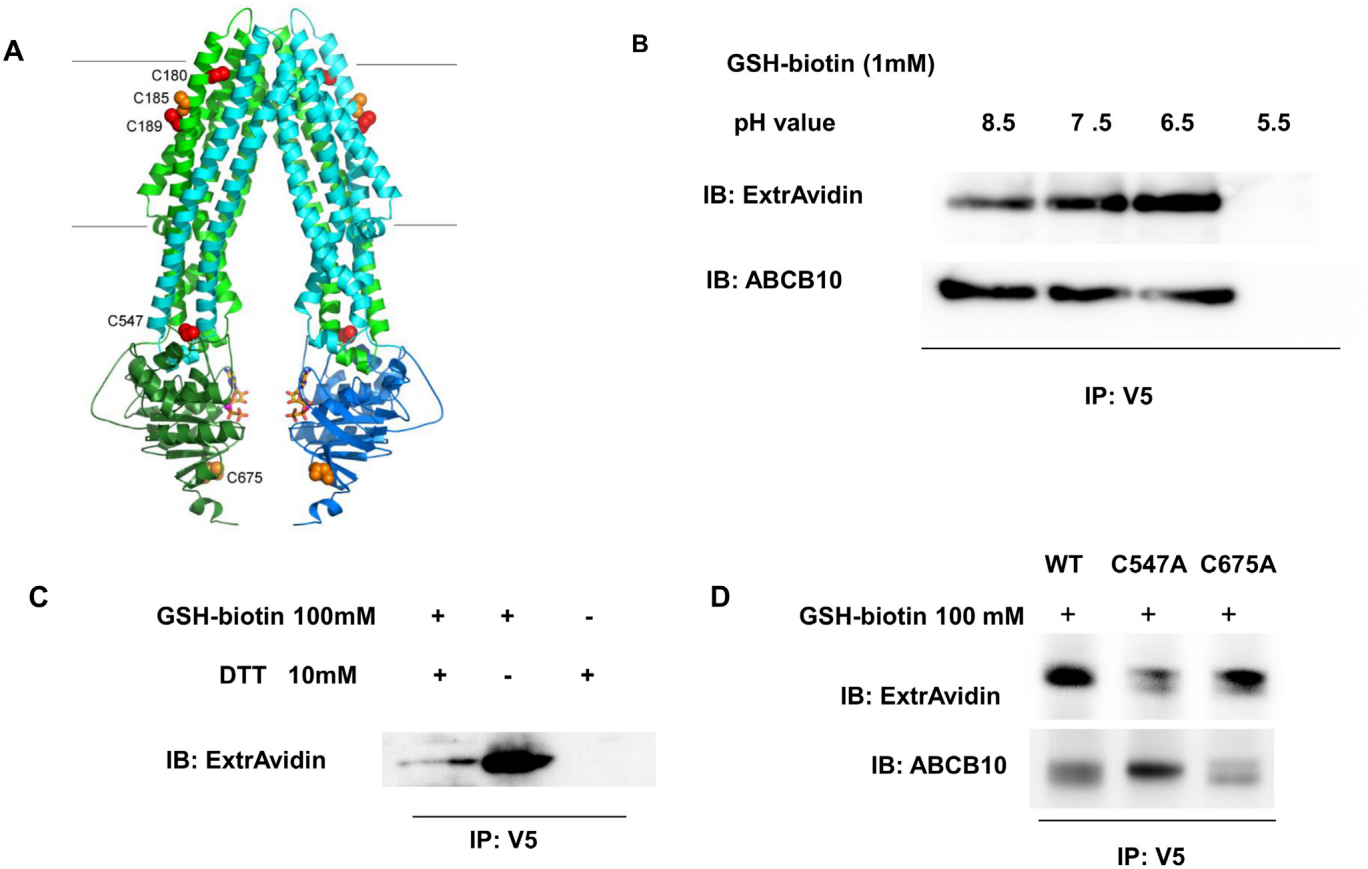
ABCB10 is glutathionylated and contains exposed cysteine residues. A) The structure of human ABCB10 (pdb:4ayx). The trans-membrane domains of the two chains are shown in green and cyan and the nucleotide binding domains (NBD) are shown in dark green and dark blue. The three conserved cysteines, Cys215/Cys180 (human/mouse numbering), Cys224/Cys189 and Cys582/Cys547 are shown in red. The residues of the human sequence that are cysteines in the mouse sequence (Ala220/Cys180 and Thr710/Cys675) are shown in orange. The residue numbering on the figure is for the mouse sequence. The approximate extent of the inner mitochondrial membrane is shown with two black lines. B) SMPs containing murine ABCB10 transfected in HEK293 cells were incubated with 1 mM biotin-labeled GSH for 4 h followed by IP with anti-V5 antibody. Glutathionylation of ABCB10 was detected with ExtrAvdin-HRP by Western blot. Glutathionylation could be detected in pH values around physiological range. At pH 5.5, the acidic environment precipitated proteins, which impeded detection of glutathionylated or total ABCB10. C) The reducing agent DTT at 10 mM prevented the glutathionylation of ABCB10 in SMPs (the detection was performed as in B, with ExtrAvidin-HRP after ABCB10 immunoprecipitation). D) Glutathionylation of ABCB10 was tested in two ABCB10-V5 mutants: Cys547 and Cys675 as in panel C, by detecting GSH-biotin within the ABCB10-V5-immunoprecipitate with ExtrAvidin by Western blot. Total ABCB10 within the immunoprecipitate was detected by probing the membrane with an ABCB10 antibody, to correct for differences in expression/immunoprecipitation efficiency caused by the mutations.

GSH/GSSG can modulate protein activity through gluthationylation of certain residues. In this regard, previous studies report that glutathionylation of Cys-1344 of the ABC protein, CFTR, inhibits its channel activity [36]. Therefore, determining cysteine residues orientation in ABCB10 structure could be helpful to understand whether ABCB10 cysteine gluthationylation is a feasible mechanism by which GSH/GSSG regulates ABCB10 ATP binding and hydrolysis activity. Based on the previously published crystal structure of human ABCB10, we have modeled mouse ABCB10 (Fig 7). Mouse ABCB10 has five cysteines in the mature protein sequence (Cys180, Cys185, Cys189, Cys547 and Cys675). Three cysteines are conserved in the mature protein from mouse to human: Cys215 (human) aligns with Cys180 (mouse); Cys 224 (human) aligns with Cys189 (mouse) and Cys582 (human) aligns with Cys547 (mouse). These conserved residues are shown in red in the human ABCB10 structure (Fig 7). The mouse sequence has two additional cysteines in the folded protein (Cys185 and Cys675), which align with human Ala220 and Thr710 and are represented in orange (Fig 7). For mitochondrial matrix GSH and GSSG to directly affect ABCB10 function, it would be necessary for the residues that are modified by the GSH/GSSG ratio to be in the matrix. Cys180, Cys185 and Cys189 are all at the apex of the protein. Cys180 is at the interface between the inner-membrane and the inter-membrane space. Cys185 and Cys189 face into the inner-membrane, not into the protein. Cys180 faces into the trans-membrane helix bundle space. None of these residues would be exposed to the mitochondrial matrix and would likely require an interaction with a membrane protein and/or intermembrane space protein attached to the membrane to be modified.

Cys547 (mouse numbering) lies at the top of the helical subdomain of the NBDs and is solvent exposed in both the open inwards and open outwards conformations. This cysteine lies near the interface between the NBD and the coupling helix from the other chain. A modification here could potentially interfere with the interactions between the NBDs and the coupling helices in a way that could be compatible with the approximately 2–5 fold change in ATP hydrolysis and binding activity induced by GSH and GSSG respectively and reported in this study.

Modification of Cys675 in the mouse sequence (T710 in the human sequence) by glutathionylation would not directly interfere with the two NBDs coming together to form closed conformation, as this position is not part of the NBD dimer interface. This residue would remain solvent exposed and on the surface of the protein in the open inwards or open outwards conformations. However, it is possible that modification of this cysteine could modulate the ATPase function by a more indirect route, not involving a direct interference of the NBDs coming together. In all, ABCB10 harbors exposed cysteine residues in ABCB10 that could potentially be glutathionylated and regulate ATP binding/hydrolysis activity. Consistent with ABCB10 structure, we detected biotin labeling by Western blot using Extravidin-HRP in immuno-precipitated ABCB10 from SMPs treated with GSH-biotin (Fig 7B and 7C). Two different results confirmed the specificity of this glutathionylation: the first was the increase of ABCB10 glutathionylation when SMPs treatments with GSH-biotin were performed at lower pH values (as di-sulfide bond formation and thus glutathionylation are decreased at basic pH values) (Fig 7B). The second was the lack of ABCB10 glutathionylation in the presence of the reducing agent DTT, which prevents di-sulfide bond formation (Fig 7C). In order to determine the cysteine residue(s) responsible for glutathionylation, we mutated the matrix exposed ones to alanine (Cys547 and Cys675) (Fig 7A) and tested whether these mutations abrogated ABCB10 glutathionylation. While mutating Cys675 did not have an effect, mutation of Cys547 impeded ABCB10 glutathionylation, as shown by the absence of specific GSH-biotin signal within ABCB10 (Fig 7D). Interestingly, glutathionylation of this cysteine could potentially interfere with the interactions between the NBDs and the coupling helices, which could alter ATPase activity. Furthermore, this Cys547 is also conserved in human ABCB10. In all, these data suggest that Cys547 can potentially be an important site for ABCB10 post-translational regulation of its ATPase activity.

## Discussion and Conclusions

This report describes the use of 8-azido-ATP to define the ATP binding and hydrolysis properties of ABCB10. We validated the specificity of the 8-azido-ATP photolabeling approach by performing competition experiments in the presence of increasing concentrations of other nucleotides, such as ATP, ADP, AMP, GTP, TTP, CTP. We found that ABCB10 photolabeling was markedly decreased in the presence of ADP and ATP, to a lower extent by GTP, TTP or CTP and unaffected by AMP, as expected if the labeling was specific. Regarding the competition by GTP, CTP and TTP, it was already shown that they can compete with ATP for binding in other ABC proteins [30].

In some ABC transporters, ATP binding was found to be stabilized by hydrogen bond interactions of the oxygen atoms from the β- and γ-phosphates of ATP with the conserved glycine and lysine residues in the Walker A motif [11]. Consistent with this, our results show that mutation of ABCB10 Walker A conserved residues, either G497A or K498R, decreased 8-azido-ATP- γ-biotin signal, likely as a result of decreased ATP binding (rather than a result of increased ATP hydrolysis). This result explained as a decrease in ATP binding is consistent with the recent analysis of ABCB10 structure, showing that oxygen atoms from the beta phosphate might form hydrogen bonds with the Walker A glycine and lysine residues [6]. A similar result was obtained when characterizing MDR1/ABCB1, another ABC transporter protein that belongs to sub-family B. This study shows that the mutation of lysine in the Walker A motif in both MDR1 NBD monomers rather than in one monomer decreased ATP binding affinity [12]. However, a different report showed that the ATP binding was not affected when one or both MDR1 NBD monomers had the conserved lysine mutated, while the transport activity was still impaired. This study implied that these mutations were reducing ATP hydrolysis activity while not affecting binding [17]. Thus, it remains possible that these glycine and lysine residues in ABCB10 are also involved in ATP hydrolysis. However, we detected decreased biotin signal in ABCB10 harboring mutations in these residues, which suggests that their requirement for ATP hydrolysis cannot be as strong as for ATP binding, as it is unlikely that these mutations would ameliorate ATP hydrolysis activity.

The mutation of the second glycine residue (G551D) in the C-loop motif has been identified in the CFTR gene of cystic fibrosis patients, showing that it is functionally important [16]. A study of the TAP protein found that mutation of glycine in the C-loop motif impaired peptide transport due to the decreased ATP hydrolysis, while the ATP binding capacity was not affected [15]. In the Walker B motif, the conserved glutamate residue immediately following the end of the motif is an important catalytic residue for ATP hydrolysis, but it is unlikely a residue that could determine ATP binding [13]. In ABCB10, we found reduced ATP hydrolysis activity in either the mutation of the second glycine in the C-loop motif or the mutation of the glutamate residue in the Walker B motif (G602V, G602D, or E624Q). Indeed, a recent study confirmed that the same mutation of glutamate to glutamine immediately after the Walker B motif in the purified and reconstituted human ABCB10 protein completely inhibits ATP hydrolysis activity, which was measured using a different approach [6]. These results show that the role of these residues in ABCB10 is equivalent to those in other ABC transporters. Thus, our study confirms that the C-loop and the glutamate immediately after the Walker B motif are important for ABCB10 ATP hydrolysis activity. Of interest some of the mutations caused an increase in the total amount of ABCB10 protein detected. These differences might be pointing to changes in conformation that might be increasing pulling down efficiency by immuno-capture or even increased stability of the protein, which are currently under investigation.

As ABCB10 is an ABC transporter, we expect that if a molecular entity increases ABCB10 ATP hydrolysis, it could be a potential substrate transported by ABCB10 and/or a potential modulator of its transport function. This means that our approach can be used to support a molecular entity as an ABCB10 substrate candidate. This is relevant, as no substrates for ABCB10 have been determined so far. ABCB10 was shown to be required for proper hemoglobin synthesis [1]. Given that ABCB10 is a transporter with the NBD facing the matrix, it was hypothesized that ABCB10 could be the unidentified ALA (heme precursor) exporter, as a direct proof of ALA transport by SLC25A38 has not been shown yet [37]. The ALA hypothesis was recently supported by the finding that ALA supplementation could rescue some defects caused by partial ABCB10 down-regulation in cardiac cell lines [34]. However, it is unknown whether down-regulation of ABCB10 was leading to decreased ALA export outside the mitochondria or decreased ALA production due to increased oxidative stress and an alteration of mitochondrial function upstream of succinyl-CoA synthesis. Our results showing that different ALA concentrations do not alter 8-azido-ATP-γ-biotin labeling in ABCB10 do not support a role of ABCB10 directly transporting dALA. The only scenario by which ABCB10 could be exporting ALA without changing the γ-biotin signal from the 8-azido-ATP bound to ABCB10 would be by promoting the exact same fold increase in ATP binding and hydrolysis activity. Thus, there is the possibility that cells with reduced ABCB10 expression might have a defect in ALA production rather than a specific defect in ALA export capacity. Another possibility could be a mitochondrial bioenergetic defect that would prevent efficient addition of iron to protoporphyrin IX and therefore prevent proper ferrochelatase activity, the latter more sensitive to oxidative stress than other heme biosynthesis enzymes [38, 39]. This defect in ALA production or in addition of iron to protoporphyrin IX might be caused by increased oxidative damage to ABCB10 deficient mitochondria, as suggested by data demonstrating that heme synthesis can be partially restored by antioxidant treatments in ABCB10 knock-out erythroid cells [2]. However, further experiments need to be done to address this possibility.

The mitochondrial electron transport chain is a major site of superoxide generation. This superoxide can be dismutated to H_2_O_2_ by superoxide dismutase 2, located in the mitochondrial matrix. H_2_O_2_ is then detoxified via mitochondrial glutathione peroxidases at the expense of turning GSH into GSSG and thus avoiding H_2_O_2_ diffusion to other cellular compartments. GSSG can be reduced back to GSH via the mitochondrial glutathione reductase (using NADPH) or could potentially be transported out of mitochondria (although the latter is controversial) [40]. By this export, the GSH-determined reducing mitochondrial environment could be maintained independently of glutathione reductase activity or the NADPH/NADP redox pair. It is important to note that GSH concentration inside mitochondria is around 10mM, and the ratio of GSH:GSSG is 20:1 to 40:1 [41, 42]. We therefore examined the effect of GSH or GSSG on the 8-azido-ATP binding or hydrolysis in our model. And we found that GSSG actually triggered 8-azido-ATP hydrolysis while GSH decreased 8-azido-ATP binding. According to the fact that an ABCB10 substrate could also decrease 8-azido-ATP-biotin labeling, we could conclude that GSSG would be one of the potential substrates of ABCB10 and that the transport of GSSG out of mitochondria could maintain the reducing (high GSH) mitochondrial environment. This could explain the anti-oxidant role of ABCB10. But this result is not sufficient to proof this hypothesis and it should be further confirmed by a transport assay. Indeed, an alternative hypothesis would be that GSSG is an activator/regulator of ABCB10 ATP hydrolysis activity rather than a transported substrate. Given that ABCB10 activity is required to protect from oxidative stress, it would make physiological sense that high GSSG in the mitochondrial matrix was the signal to sense increased oxidative stress, thus activating ABCB10 mediated protection. In this regard, we investigated whether ABCB10 was glutathionylated and the presence and orientation of cysteine residues in ABCB10 that could be potentially glutathionylated. This glutathionylation could represent the molecular mechanism by which GSH and GSSG modulate ABCB10 ATP hydrolysis activity.

For the ATPase reaction to occur, the two NBDs would have to come together to form a close packed open outwards arrangement, as seen in the MsbA and Sav1866 structures. Residues involved in large changes in ATPase activity would be expected to lie at the interface between the NBDs or the TMDs, or to lie in a region that is important for activity or changes in conformation. None of the cysteine residues of ABCB10 lie in this region. Three out of the 5 cysteines are exposed to the inner membrane but closer to the inter-membrane space, and are relatively far from the NBD. The modification of these 3 cysteines would likely require an interaction with a membrane protein (integral and/or attached). The fourth cysteine (Cys547) lies near the interface between the NBD and the coupling helix from the other monomer and its modification could potentially interfere with the interactions between the NBDs and the coupling helices. Therefore, this is a feasible hypothetical mechanism by which glutathionylation could alter ATPase activity. The fifth cysteine, Cys675, is located in the NBD in a region outside the NBD dimer interface. Therefore, a modification in this cysteine could potentially affect ATPase by an indirect mechanism that would not directly interfere with the NBDs coming together, a step required for ATP hydrolysis. In this regard, we identified that Cys547 is the residue responsible for glutathionylation mediated by GSH, whereas mutation of Cys675 to alanine did not have an effect on ABCB10 glutathionylation. Given that Cys547 is a conserved residue in mice and human and is located in a relevant region of the protein, which could alter ATP binding and hydrolysis activity if this residue was modified, we propose that this residue might have a relevant role modulating ABCB10 ATPase activity *in vivo*.

Altogether, this study demonstrates that the 8-Azido-ATP approach can be used to provide evidence to support and/or reject certain molecular entities as hypothetical substrates transported by ABCB10. In addition, it can be used to identify modulators and the effect of certain mutations in ABCB10 ATP binding and hydrolysis activity. Indeed, this methodology allowed us to identify that GSH/GSSG ratio, likely through glutathionylation, can modulate ABCB10 activity. Moreover, we provided preliminary evidence that ALA might not be a substrate directly exported by ABCB10.
